# Behavioural responses of a cold-water benthivore to loss of oxythermal habitat

**DOI:** 10.1007/s10641-022-01335-4

**Published:** 2022-09-28

**Authors:** Tazi H. Rodrigues, Andrew J. Chapelsky, Lee E. Hrenchuk, Graham R. Mushet, Lauren J. Chapman, Paul J. Blanchfield

**Affiliations:** 1 IISD Experimental Lakes Area, 111 Lombard Avenue, Suite 325, Winnipeg, MB R3B 0T4 Canada; 2 Department of Biology, McGill University, 1205 Dr Penfield Ave, Montreal, QC H3A 1B1 Canada; 3 Fisheries and Oceans Canada, 501 University Crescent, Winnipeg, MB R3T 2N6 Canada; 4 Department of Biology, Queen’s University, 116 Barrie Street, Kingston, ON K7L 3N6 Canada

**Keywords:** Fish, Lake whitefish, Climate, Dissolved oxygen, Telemetry

## Abstract

Climate-driven declines in oxythermal habitat in freshwater lakes can impose prolonged constraints on cold-water fishes sensitive to hypoxia. How fish cope with severe habitat limitations is not well understood, yet has implications for their persistence. Here, we use acoustic-positioning telemetry to assess seasonal habitat occupancy and activity patterns of lake whitefish (*Coregonus clupeaformis*), a cold-water benthivore, in a small boreal lake that regularly faces severe oxythermal constraints during summer stratification. During this stratified period, they rarely (< 15% of detections) occupied depths with water temperatures > 10 °C (interquartile range = 5.3–7.9 °C), which resulted in extensive use (> 90% of detections) of water with < 4 mg L^−1^ dissolved oxygen (DO; interquartile range = 0.3–5.3 mg L^−1^). Lake whitefish were least active in winter and spring, but much more active in summer, when only a small portion of the lake (1–10%) contained optimal oxythermal habitat (< 10 °C and > 4 mg L^−1^ DO), showing frequent vertical forays into low DO environments concurrent with extensive lateral movement (7649 m d^−1^). High rates of lateral movement (8392 m d^−1^) persisted in the complete absence of optimal oxythermal habitat, but without high rates of vertical forays. We found evidence that lake whitefish are more tolerant of hypoxia (< 2 mg L^−1^) than previously understood, with some individuals routinely occupying hypoxic habitat in winter (up to 93% of detections) despite the availability of higher DO habitat. The changes in movement patterns across the gradient of habitat availability indicate that the behavioural responses of lake whitefish to unfavourable conditions may lead to changes in foraging efficiency and exposure to physiological stress, with detrimental effects on their persistence.

**Supplementary Information:**

The online version contains supplementary material available at 10.1007/s10641-022-01335-4.

## Introduction

Anthropogenic warming of the Earth’s atmosphere is having profound effects on freshwater lakes. For north temperate lakes, some of these effects include warmer water temperatures, stronger and longer periods of stratification, shorter duration of ice cover, and widespread increases in hypoxia (O’Reilly et al. 2015; Woolway et al. 2020; Jane et al. 2021). Collectively, these climate-induced changes in lake thermal structure have the potential to greatly alter both the quantity and quality of habitat available to fishes, as specific water temperature and dissolved oxygen (DO) concentration criteria (i.e., oxythermal habitat) have been shown to be important in defining their habitat requirements (Gibson and Fry 1954; Magnuson et al. 1979; Coutant 1987; Arend et al. 2011). This is particularly problematic for lacustrine fishes that require cold, well-oxygenated water, such as those in the family Salmonidae (including salmon, trout, char, grayling, and whitefishes), whose preferred thermal habitat can become greatly reduced during summer stratification and largely restricted to deep hypolimnetic waters (Plumb and Blanchfield 2009; Magee et al. 2019). During this stratification, the hypolimnion is physically isolated from the atmosphere, and oxygen renewal can be negligible while respiration and decomposition—particularly at the lake bottom—continue to deplete DO (Wetzel 2001). The decline in hypolimnetic DO concentrations to levels below what fish require can severely limit, or even eliminate, appropriate oxythermal habitat available to cold-water fishes during summer (Guzzo and Blanchfield 2017; Magee et al. 2019).

Lake whitefish, *Coregonus clupeaformis* (Mitchill 1818), is an ecologically, economically, and culturally important species (Rennie et al. 2009; Pothoven 2020). It is a cold-water, benthic-reliant fish whose optimal thermal range for growth has been estimated to lie between 10 and 14 °C (Christie and Regier 1988). Typically, lake whitefish are found in deep lacustrine habitat, except during fall spawning when they move to shallower areas (Anras et al. 1999; Gorsky et al. 2012). The hypoxia tolerance of adult lake whitefish in nature is understudied, but research on several related species has found DO avoidance thresholds of 2 mg L^−1^ for vendace (*Coregonus albula* Linnaeus 1758) (Elliott and Bell 2011), 3 mg L^−1^ for cisco (*Coregonus artedi* Lesueur 1818) (Jacobson et al. 2010), and 4–6 mg L^−1^ for lake trout (*Salvelinus namaycush* Walbaum 1792) (Plumb and Blanchfield 2009). Temperature-based models suggest that the geographical range of lake whitefish, which currently extends from northern Canada (near Cambridge Bay, Nunavut) south to the Great Lakes basin and northeastern United States (Rogers 2008; Page and Burr 2011), will shift northward as climate change drives extirpation in warmer lakes along the southern edge of their range and extends habitat availability into colder (but warming) northern lakes (Poesch et al. 2016; Biswas et al. 2017). Indeed, roughly one third of former lake whitefish populations in inland Wisconsin lakes may already be extirpated, likely caused by a combination of introduced rainbow smelt (*Osmerus mordax* Mitchill 1814) populations and declining oxythermal habitat (Renik et al. 2020), and more than 70% of cisco populations in this state are predicted to be extirpated by 2100 (Sharma et al. 2011).

Cold-water benthivores, like lake whitefish, may be especially sensitive to declines in oxythermal habitat because reductions in area of optimal lake bottom are proportionally greater than reductions in lake volume for a given set of oxythermal constraints, which can disproportionately diminish foraging opportunities. Furthermore, the preferred prey taxa of lake whitefish, chironomids (family Chironomidae) and chaoborids (*Chaoborus spp.*), include species that are highly tolerant of hypoxia and anoxia (Pinder 1995; Stratton and Kesler 2007). As such, lake whitefish access to key invertebrate prey items is likely to become more physiologically challenging as their habitat becomes further constrained and they are forced to feed either within diminishing optimal oxythermal habitats or under suboptimal habitat conditions. Recent studies have highlighted variability in thermal habitat selection by lake whitefish among lakes (e.g., Gorsky et al. 2012; Challice et al. 2019), but because hypolimnetic DO was presumably not limiting in these studies (> 7 mg L^−1^; Challice et al. 2019), habitat selection under both temperature and DO constraints is not known.

Dissolved oxygen concentrations can be highly variable in aquatic systems (Soitamo et al. 2001), so many fishes must have effective responses to hypoxia to survive. Hypoxia is most frequently defined as < 2 mg L^−1^ (Hrycik et al. 2017), although low oxygen may start to affect fish even at higher concentrations depending on the species (Hrycik et al. 2017). For example, Evans (2007) modelled that the power output of juvenile lake trout diminished markedly as ambient DO concentrations declined below 7 mg L^−1^ at standard summer hypolimnetic temperatures for this species (4–8 °C). Brief foraging forays into the hypoxic hypolimnion have been observed for several fish species (Rahel and Nutzman 1994; Taylor et al. 2007). Yellow perch (*Perca flavescens* Mitchill 1814), for instance, were observed to shift their diet towards pelagic prey during stratification, with forays into the hypoxic hypolimnion mostly occurring when the hypolimnion was small or highly concentrated with chironomids (Roberts et al. 2009). While it is expected that various fish species can tolerate short exposures to suboptimal oxythermal conditions, how fish respond behaviourally and the habitat choices they make when confronted with prolonged and severe reductions in oxythermal habitat remains an important question to address.

This study examined the behavioural response of lake whitefish to changing oxythermal conditions to (a) characterize lake whitefish habitat use in relation to varying amounts of oxythermal habitat constraint across one year, (b) determine the behavioural responses (activity) and habitat choices made by lake whitefish when coping with the complete loss of oxythermal habitat, and (c) provide evidence for adult lake whitefish hypoxia tolerance in the wild. We followed the seasonal changes in the spatiotemporal distribution of lake whitefish in a small boreal lake that experiences considerable seasonal variability in the amount of available oxythermal habitat, including complete loss of optimal oxythermal habitat (temperature > 10 °C and DO < 4 mg L^−1^) in summer and extended periods of winter hypoxia (< 2 mg L^−1^). We combined passive acoustic positioning telemetry data and habitat profiles collected during a year-long period to examine the effect of oxythermal habitat conditions on lake whitefish behaviour at daily and seasonal scales. Lastly, we used long-term data to demonstrate the duration and frequency of oxythermal constraints faced by this population of lake whitefish.

## Methods

### Study site

**Fig. 1:**
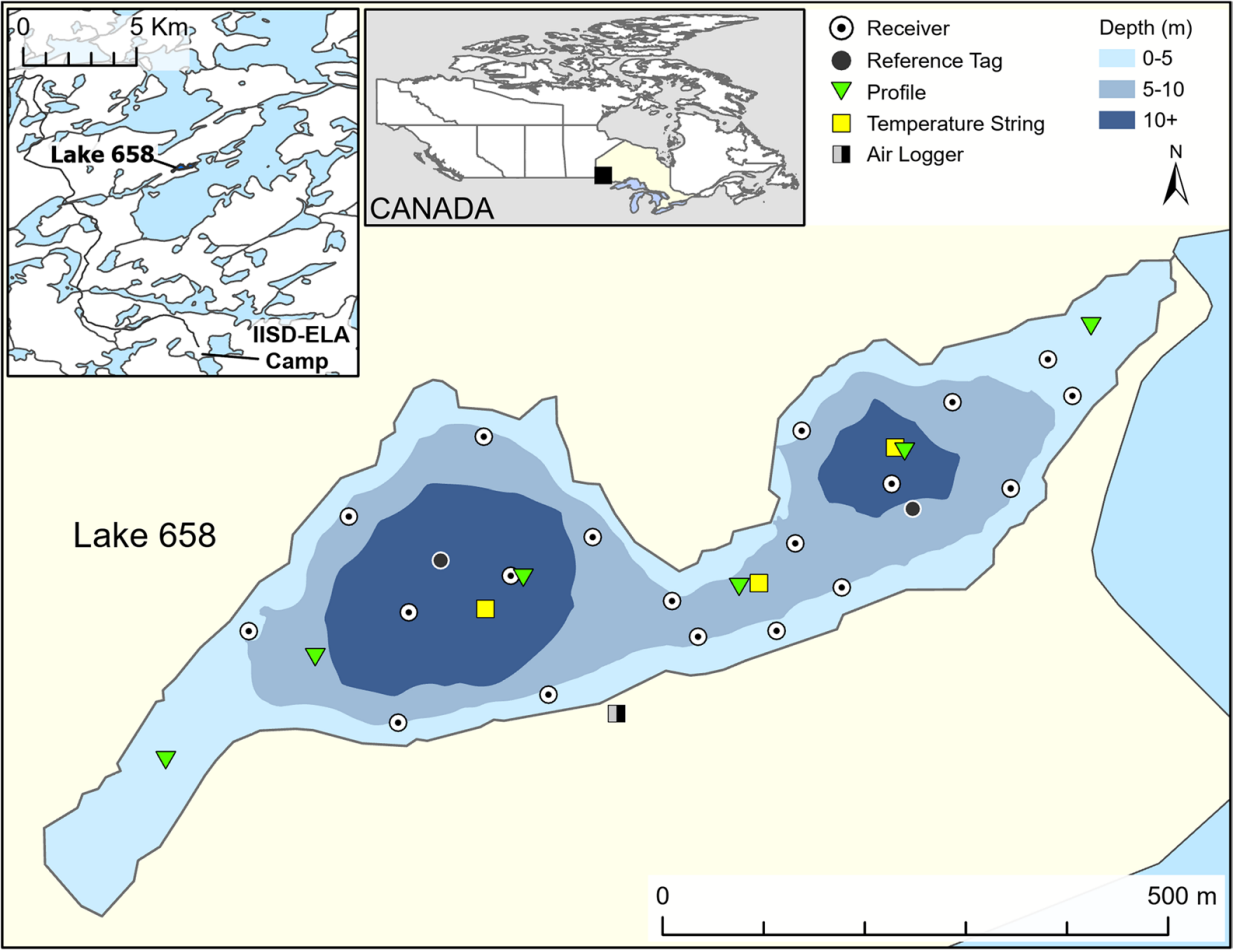
Bathymetric map of Lake 658 showing the array of telemetry receivers (circles) for fish positioning, temperature profile locations (squares), and oxygen profile locations (triangles). The location of Lake 658 within the IISD Experimental Lakes Area is shown in the top left inset and its location in Canada is shown in the top middle inset

Lake 658 is a small (8.4 ha), double-basin headwater lake that is part of the IISD Experimental Lakes Area (IISD-ELA hereafter)—an area in northwestern Ontario set aside for research on freshwater lakes and their watersheds (Blanchfield et al. 2009a). The West and East basins of Lake 658 have maximum depths of 13.3 m and 11.3 m, respectively, and are separated by a 6 m deep saddle (Fig. 1). The lake is dimictic, with turnover typically occurring in May and October (Small and Hintelmann 2014), although mixing is not always complete. This remote lake has been the site of a whole-ecosystem experiment where a small amount of enriched isotopic mercury was added to the lake and watershed over a period of seven years (2001–2007; Blanchfield et al. 2022). The lake is closed to fishing.

In addition to lake whitefish, fish species in Lake 658 include northern pike (*Esox lucius* Linnaeus 1758), white sucker (*Catostomus commersonii* Lacepède 1803), yellow perch, and various minnow species (*Notropis heterolepis* Eigenmann and Eigenmann 1893, *N. hudsonius* Clinton 1824, *Pimephales promelas* Rafinesque 1820). Lake 658 is attached to a larger neighbouring lake, Winnange Lake (2613 ha; Blanchfield et al. 2014), from which its lake whitefish population is likely derived. It is probable that the two populations have been separated for a long time (Blanchfield unpublish. data), and a fence between the two lakes has prevented movement of fish since 2001. The population size of adult lake whitefish in Lake 658 is small (estimated to be an average of 134 individuals for 2000–2015; M. D. Rennie, personal communication) and is largely comprised of large-bodied (mean fork length = 531 mm, SD = 52 mm; mean weight = 2245 g, SD = 583 g), old (median age = 17 y, range = 3–38 y) individuals, based on gill net captures (Blanchfield et al. 2022). The macroinvertebrate community includes profundal and littoral chironomids, chaoborids, mayflies (Order Ephemeroptera), and amphipods (Order Amphipoda).

### Limnological data

Detailed water temperature and DO data were collected during the period that lake whitefish habitat use was monitored in Lake 658 (May 2015–May 2016). Temperature profiles were taken every hour throughout the year by strings of HOBO Pendant Temp/Light loggers (64 k model UA-002–64, Onset Computer Co., Cape Cod, MA; ± 0.53 °C from 0 to 50 °C) at 0.5-m depth and then at 1-m intervals from 1 to 6 m (Saddle), 10 m (East basin), or 13 m (West basin) (Fig. 1). Oxygen profiles were collected approximately every 2 weeks from May 11 2015 to November 5 2015, and once on March 17 2016, with a YSI ProODO handheld meter (range = 0–50 mg L^−1^, accuracy ± 0.1 mg L^−1^ for 0–20 mg L^−1^; YSI Inc., Yellow Springs, OH). DO concentrations were measured at 1-m intervals from the surface to the lake bottom at the deepest points in the West and East basins and at the centre of the saddle.

Historical collections of water temperature and DO profile data have occurred throughout the open water seasons (May–October) of 2000–2010 and in 2014. All profiles were collected at the West basin centre buoy (Fig. 1) at 1-m depth increments from lake surface to bottom (~ 13 m) according to standard protocols at IISD-ELA. From 2000 to 2009, temperature profiles were collected approximately every 2 weeks with Flett Research Mark II digital telethermometers. In 2010, these data were collected monthly with the temperature sensor in the multi-parameter probe RBR XRX620 (range =  − 5–35 °C, accuracy =  ± 0.002 °C; RBR Ltd., Ottawa, ON). In 2014, temperature profiles were taken approximately monthly with a YSI ProODO handheld meter (range =  − 5–45 °C, accuracy =  ± 0.3 °C; YSI Inc., Yellow Springs, OH). Oxygen profile samples were collected approximately once per month during the open water seasons and once or twice per winter from 2000 to 2010, and DO data were derived with Winkler titrations performed on site at the IISD-ELA chemistry laboratory (Stainton et al. 1977). In 2014, DO profiles were collected with the YSI ProODO handheld meter. We used these data to examine longer-term trends in lake whitefish habitat availability.

### Fish telemetry

Lake whitefish were captured for transmitter implantation using short sets (< 30 min) of braided nylon gillnets (3.5″, 4.0″, or 4.5″ mesh) in early May 2015, when surface water temperature was below 10 °C. Fish were transported in a closed cooler filled with ambient lake water from the site of capture to a holding pen near to shore where they were held until surgery (< 48 h). Five adult fish (total length = 570–598 mm, mass = 1946–2417 g) were tagged with pressure-sensing transmitters (V13P, Vemco Ltd., Bedford, Nova Scotia, Canada). Surgical implantation of transmitters followed established protocols for cold-water fish species (see Blanchfield et al. 2005). All equipment was sterilized with 95% ethanol and cleaned in reverse osmosis distilled water. Once removed from the holding pen, fish were transferred to a 20 L anaesthetic bath containing 1.8 g tricaine methanesulfonate (TMS, MS-222) and 3.6 g sodium bicarbonate (NaHCO_3_) buffer, which is equivalent to 90 mg L^−1^ anaesthetic concentration. When fish overturned and did not respond to squeezing of their caudal peduncle (mean time 7.5 min), the wet weight was measured with a Mettler scale (16 000 g, accurate to 0.1 g), and total length (mm) and fork length (mm) were measured.

Surgery took place in a V-notched cradle lined with nylon mesh while a maintenance anaesthetic (0.9 g TMS, 1.8 g NaHCO_3_, 20 L water, equivalent to 45 mg L^−1^ anaesthetic concentration) was pumped over the gills. For each fish, a 2–3-cm-long incision was made in the ventral midline, anterior to the pelvic girdle. A transmitter was inserted into the intracoelomic cavity and the incision closed with two standard surgical knots tied with Monocryl (Y923H, Ethicon US, LLC). Combined, all measurements and surgeries took less than 9 min, with an average of ~ 8 min. After surgery, fish were placed in a cooler of lake water (< 10 °C) with lightly bubbling oxygen until they could maintain their balance and beat their tail when the caudal peduncle was squeezed (< 20 min). They were subsequently transferred into a floating, open-topped mesh pen and released to the lake when normal swimming behaviour had resumed.

Transmitters emitted acoustic signals that encoded depth, measured by a pressure sensor (maximum depth = 17 m, accuracy ± 1.7 m, resolution = 0.08 m; Innovasea 2021), every 60–180 s, along with a unique identifier for each fish. Upon detection by an array of underwater omnidirectional acoustic receivers (VR2W, 69 kHz), acoustic data (fish ID and depth) were logged and timestamped. A total of 19 receivers were deployed in a Vemco Positioning System (VPS) array throughout Lake 658 to optimally determine lake whitefish positions (Fig. 1). Receivers ranged in depth from 2.0 to 12.1 m and were attached to a 1.3-cm braided rope using cable ties ~ 2 m above the cinder block anchor, which was ~ 1–2 m above the lake bottom depending on lake depth. A subsurface float was attached at least 1.5 m below the surface to keep tension on the anchor rope, with an extra 2–3 m of slack rope between subsurface and surface floats. Data were downloaded from the receivers in May, September, and November 2015 and May 2016. All receiver data, along with pertinent water temperature data, were sent to Vemco Ltd. for processing and determination of the spatial positions of lake whitefish (see below).

### Data processing and analysis

All analyses were conducted in RStudio (R Core Team 2022). Data collected by the temperature strings during May 2015–May 2016 were linearly interpolated to every 0.1 m between 0.0 and 13.0 m for each hour that data were collected, and DO data were linearly interpolated to every 0.1 m and every day during the study period with the “forecast” package (v. 8.13; Hyndman et al. 2021). Daily mean temperatures were subsequently calculated for every 0.1 m to match the resolution of the DO data for calculations of oxythermal habitat availability. Although only one DO profile was taken during the winter (March 2016), we used linear interpolation throughout the study period under the assumption that oxygen concentrations would not increase under ice. Historical temperature and DO data were interpolated to every 0.1 m with the “forecast” package (Hyndman et al. 2021) between 0.0 and 13.0 m for each day on which data were collected and then for each day during the years (2000–2010, 2014–2015).

Based on the thermal niche for lake whitefish defined by Christie and Regier (1988) of 10–14 °C, we used 10 °C as the upper threshold for optimal thermal habitat and 14 °C as the upper threshold for usable habitat. Recent research showing wild lake whitefish prefer primarily < 14 °C (Challice et al. 2019) supports the use of these temperature thresholds. Optimal and usable DO thresholds for lake whitefish have not been as well defined as for other closely related coldwater species, such as lake trout. A generalized threshold of 3 mg L^−1^ has been suggested for coldwater fishes by Jacobson et al. (2010) and has subsequently been used in several oxythermal habitat assessments for coregonids (e.g., Magee et al. 2019; Grow et al. 2022). Here, we used the slightly higher threshold of 4 mg L^−1^ for optimal oxygen habitat, which corresponds to the lower bound of optimal oxygen habitat for adult lake trout in nearby lakes at IISD-ELA (Plumb and Blanchfield 2009). Thus, usable oxythermal habitat for lake whitefish was the portion of the water column (and lake bottom) contained between the 14 °C and 2 mg L^−1^ DO isoclines (i.e., < 14 °C and > 2 mg L^−1^ DO), whereas optimal oxythermal habitat was bounded by the 10 °C and 4 mg L^−1^ DO isoclines. The daily average temperatures and daily DO values at each depth were used to calculate the depth in the water column of 14 °C, 10 °C, 4 mg L^−1^ DO, and 2 mg L^−1^ DO, from which we calculated the daily extent of oxythermal habitat throughout the year.

The percent lake volume and bottom area that satisfied these conditions were calculated for each day during the period of lake whitefish monitoring (2015–2016) based on bathymetric data (Sandilands et al. 2005) and isocline depths. Lake 658 has a total volume of 547 966 m^3^ and total bottom surface area of 86 227 m^2^. If water at a specific depth was > 14 °C, the volume of water and the bottom surface area above this depth were considered unusable regardless of its oxygen concentration. Water with < 2 mg L^−1^ DO was similarly considered unusable, such that only depths that satisfied both thermal and oxygen concentrations was considered usable oxythermal habitat, and likewise only water < 10 °C with > 4 mg L^−1^ DO was considered optimal habitat. Because temperature and oxygen profiles were similar across sampling sites in the lake (a median percent difference between basins of ~ 5%), we used only data from the deepest basin (West; Fig. 1) to derive the oxythermal conditions of Lake 658. For the historical data (2000–2010 and 2014–2015), we used the same criteria to calculate the minimum extent of optimal and usable oxythermal habitat per year, the number of days without optimal or usable habitat per year, and the proportion of lake volume that satisfied the parameters for optimal and usable habitat per year.

Raw acoustic telemetry data downloaded from the receivers, which included a date-timestamp, fish ID, and depth for each detection, were time-corrected with synctag detections to account for clock drift using the Vemco User Environment (VUE; Vemco Ltd.) and subsequently imported into RStudio. Our array of 19 receivers allowed for complete spatial coverage of Lake 658 (Fig. 1), but also resulted in many duplicate detections of fish because of the overlapping detection range of the receivers. We filtered the data to exclude all detections that were less than 60 s apart from the same fish (assumed duplicate detections), occurred at depths greater than 14 m, or did not correspond to a known fish ID. We used this dataset (856 606 detections) to examine habitat use and vertical movements of lake whitefish in relation to changing oxythermal constraints. Spatial data from the acoustic telemetry array processed by Vemco Ltd. provided coordinates of fish positions. Because of the extensive receiver network for the size of the lake (Fig. 1), we included all spatial data available in our estimates of daily movement rates. Based on the threshold set by Cott et al. (2015), we calculated the percentage of detections with unitless hyperbolic positioning error (HPE) > 20 and subsequently decided not to filter data by HPE as they accounted for relatively few detections (4%). To account for the immediate effect of surgery on behaviour, detections collected before May 23 were removed (min. 8 days after surgery). Individual fish IDs were named LW6 through to LW10.

For each fish detection (which was a depth value at a specific date and time), we determined the corresponding hourly ambient water temperature and daily DO values, both interpolated to 0.1 m. From these data, we describe temperature and DO habitat use by tagged lake whitefish and calculate the proportion of fish detections in usable and optimal temperature and DO conditions. When both habitat parameters (temperature and DO) were combined to calculate the proportion of fish detections in usable and optimal oxythermal conditions, we calculated daily mean temperatures at depth in order to match the resolution of the DO data, which were interpolated to daily intervals because of the greater timespans between sampling events.

To compare lake whitefish behaviour and activity across a gradient of oxythermal conditions, we categorized the seasonal data into four periods of habitat constraint. In chronological order, these were moderate—when < 50% but > 10% of the lake bottom area satisfied optimal habitat conditions (May 23–June 26, 2015); high—when 1–10% of the lake bottom area satisfied optimal habitat conditions (June 27–August 30, 2015); severe—when < 1% of the lake bottom area satisfied optimal habitat conditions, including 29 days when there was no optimal habitat at all (August 31–October 8, 2015); and low—when > 50% of the lake bottom area satisfied optimal habitat conditions (October 23, 2015–May 4, 2016). Rapid change in the amount of available optimal habitat occurred during the 2 weeks associated with autumn turnover of the lake, and were excluded from these category-based analyses.

Lake whitefish habitat occupancy was assessed per day to compare among the four periods of differing oxythermal constraint. The proportions of fish detections within usable and optimal habitat were calculated for twenty consecutive days within each period of habitat constraint (May 23–June 11, 2015; July 23–August 11, 2015; September 17–October 6, 2015; and January 23–February 11, 2016; all dates inclusive). Proportions were calculated as the number of detections satisfying specific oxythermal criteria (e.g., < 14 °C and > 2 mg L^−1^) divided by the total number of detections, and were then compared among periods with a linear mixed-effects model (see below). The 20-d periods allowed clearer separation of oxythermal habitat constraints and were chosen as the first 20 days available and every 2 months thereafter, excluding November, when results were likely to be affected by spawning behaviour. These 20-d periods were used for subsequent analyses.

To assess movement rates under changing oxythermal constraints, we compared distances travelled by fish vertically (from the depth dataset) and laterally (from the positional dataset), separately. Distance travelled per day was calculated for each fish by calculating the distance between each successive pair of detections. Lateral distances were calculated using the distHaversine function in the “geosphere” package (v. 1.5.10; Hijmans 2019). To assess vertical movement, we calculated the absolute difference in depth between successive detections based on the depth data provided by the pressure transmitter. Total daily vertical and horizontal movement by individual fish were calculated separately by summing all vertical and horizontal distances travelled by individual fish each day. Because each distance calculated by change in telemetry position represents the minimum distance moved between detections, the sums represent minimum daily total distances. To account for differences in body size, we also calculated mean daily swim speed. For this analysis we divided the distance between successive detections (m) by the time between the two detections (s). We then multiplied by 60 and divided by total length of the fish to derive swim speed in body lengths per minute for each individual.

### Statistical analysis

To visualize temporal patterns in the depth, temperature, and DO of the strata that individual lake whitefish were detected in, as well as patterns in movement, we used generalized additive models (GAMs). Here, the response variable is modelled as the sum of smooth functions of the independent variables, making this approach ideal in our case because it can model non-linear responses as are often observed in time-series data (Simpson and Anderson 2009; Simpson 2018). GAMs can also be formulated to model a global (common trend) among a group (e.g., multiple individuals), while also allowing for intergroup variation by including a second term in the model that specifies group-level smooths (Pedersen et al. 2019). We used a single model for each of our response variables (depth, temperature, DO, vertical and lateral distance, and swim speed). In all cases, these models included a global smooth that modelled the response as a function of Julian day, plus a factor-smoother interaction that specified group-level (i.e., individual fish ID) smooth terms. The latter term in the model uses a penalty to draw individual smooths toward zero and is equivalent to model “GS” presented in Pedersen et al. (2019). For all five models, error distributions from either the gamma or tweedie family with a log link function were selected, and thin plate splines were used as the basis spline. Likewise, restricted maximum likelihood (REML) was used as the smoothness selection method for all models. Generalized additive models were fit using the package “mgcv” (Wood 2011; Wood et al. 2016).

To determine if the a priori defined oxythermal stress categories had a significant influence on our response variables of interest, we used the data from the consecutive 20-d periods that corresponded to each stress category. Because we had repeated measurements on individual fish, we completed this analysis using a linear mixed model framework, which enabled us to include fish ID as a random effect. Two models were initially generated: a null model using only fish ID as a random intercept, and a second model that also included category as a predictor. These models were fit using maximum likelihood and were compared using the Akaike Information Criterion (AIC) and Bayesian Information Criterion (BIC) which helped us to assess the importance of stress category as an explanatory variable. The model that included category as a predictor was refitted using restricted maximum likelihood and the significance of category was assessed using the “Anova” function (Type II) from the R package “car” (Fox and Weisberg 2019). In the case that category was significant, we used the “emmeans” package (Lenth 2022) to estimate marginal means and to test Tukey-adjusted pairwise comparisons of the category variable. All models included a first-order autoregressive error structure (AR1) to help account for potential non-independence between observations over our 20-d periods, in addition to a variance structure (varIdent, package “nlme;” Pinheiro et al. 2020) to allow for different variances among categories. Finally, response variables were log-transformed where necessary to achieve a more normal error distribution which were assessed via visual inspection of Q-Q plots and histograms. All models were fit using the package “nlme” (Pinheiro et al. 2020).

## Results

**Fig. 2:**
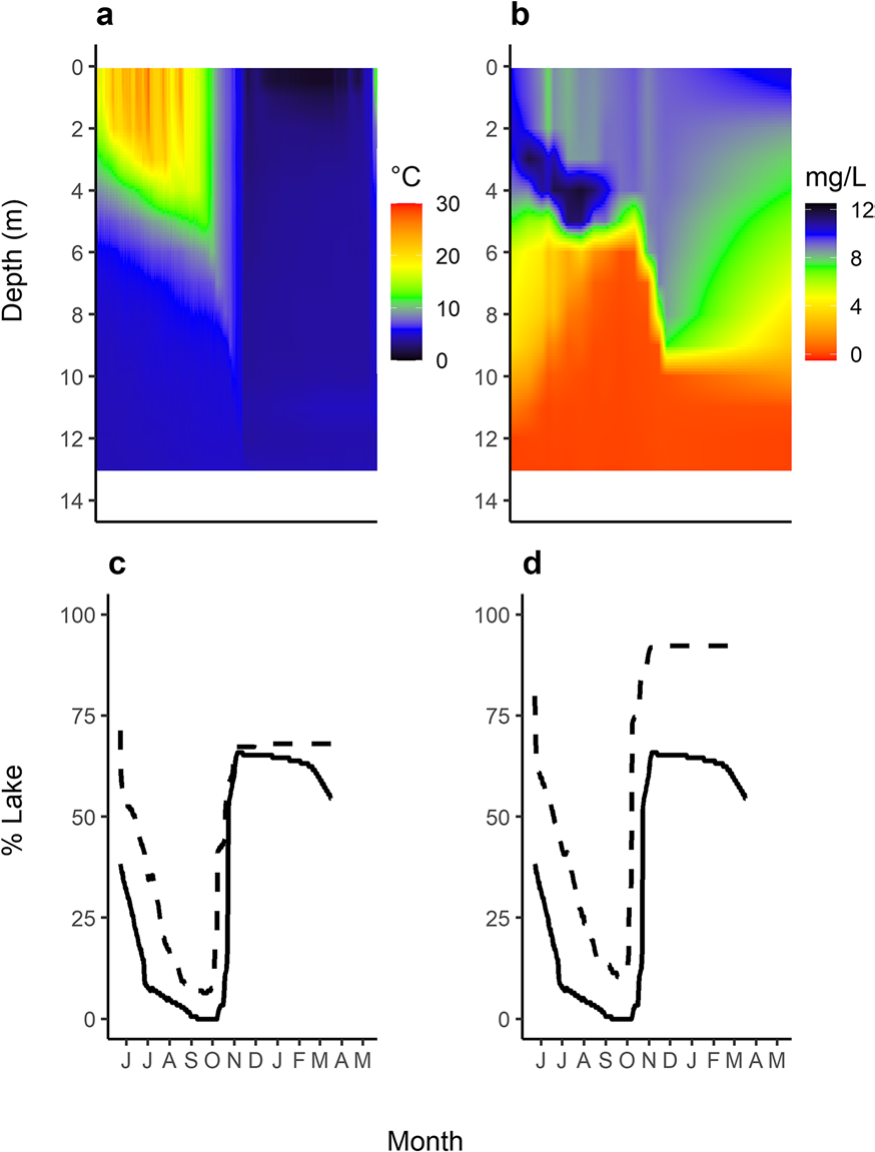
**a** Water temperature and (**b**) dissolved oxygen (DO) concentrations by depth in Lake 658 from May 2015–May 2016. The percent of (**c**) lake bottom area and (**d**) lake volume that satisfy usable (14 °C and 2 mg L^−1^; dashed line) and optimal (10 °C and 4 mg L^−1^; solid line) habitat requirements for lake whitefish

During the year-long lake whitefish tracking study, the maximum surface water temperature of Lake 658 was 28.2 °C on July 27, 2015 (Fig. 2a). Water temperatures exceeded 14 °C in at least part of the lake from the start of the study (May 23, 2015), until early fall (October 4, 2015; Fig. 2a). The 14 °C isotherm reached a depth of 4.5 m for one month in late summer (August 25–September 27, 2015) with the deepest extent of 4.7 m on September 27, 2015. The 10 °C isotherm followed a similar pattern, with water temperatures exceeding this value for the upper 5 m of the lake for most of the spring and summer before reaching its deepest extent of 6.2 m in early fall (October 14, 2015), after which the lake cooled rapidly (Fig. 2a).

The upper 5 m of the water column was well oxygenated during the stratified period (mean = 9.2 mg L^−1^), as was much of the water column following autumn turnover on October 9 (min in upper 6 m = 4.3 mg L^−1^; Fig. 2b). However, hypoxia persisted in the bottom waters throughout the study period (Fig. 2b). DO concentrations were consistently below 2 mg L^−1^ in the bottom 1.8 m of the lake and below 4 mg L^−1^ in the bottom 3.4 m (Fig. 2b). From the beginning of the study, both DO oxyclines steadily rose in the water column from the lake bottom, reaching maximum depths in early fall (5.3 m for 2 mg L^−1^ and 5.7 m for 4 mg L^−1^).

**Fig. 3:**
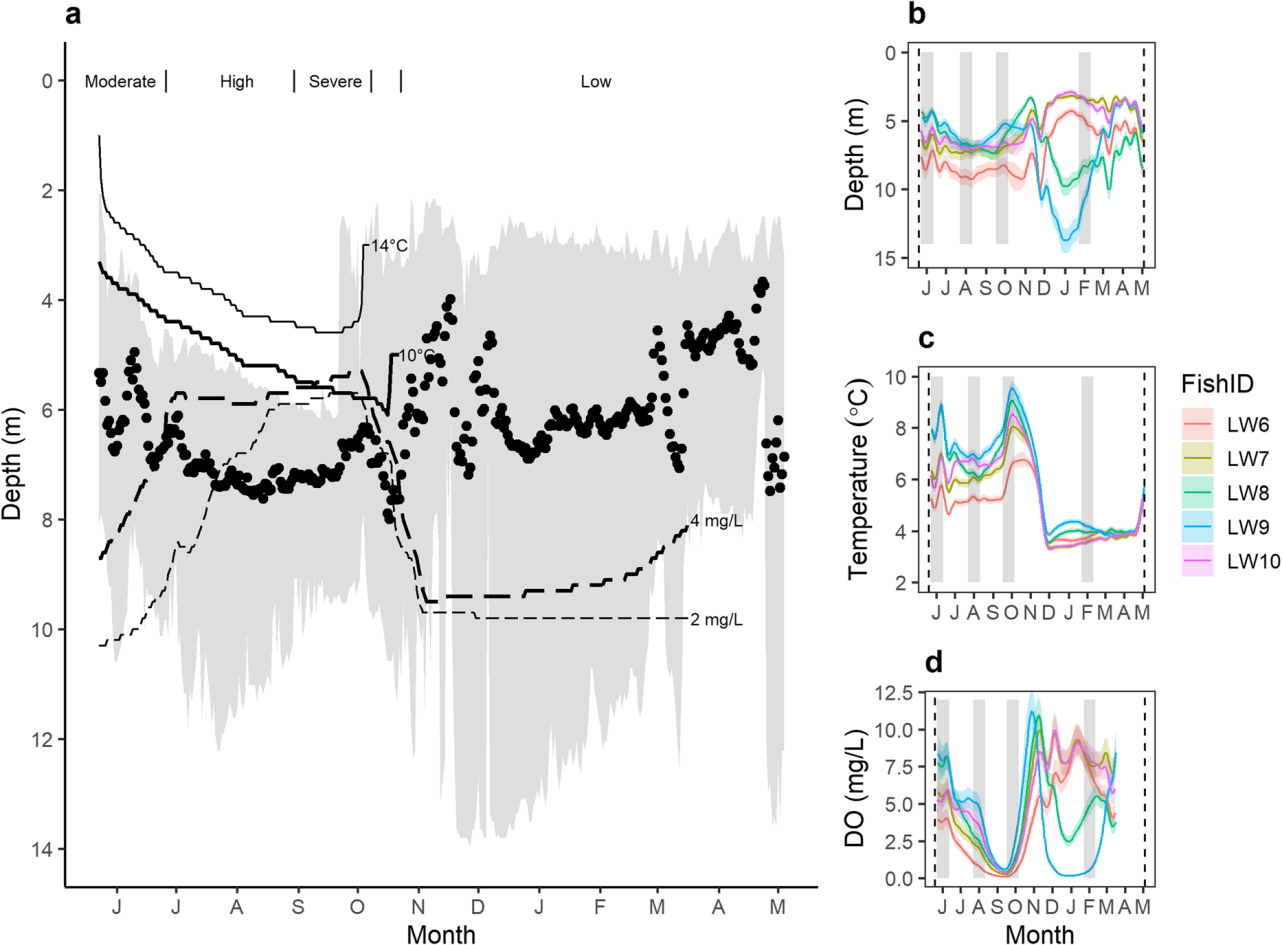
**a** Grand mean daily depths (with 2.5–97.5 percentiles; grey shading) for acoustically tagged lake whitefish in Lake 658. Oxythermal habitat boundaries are depicted for water temperature (14 °C and 10 °C) and dissolved oxygen concentrations (4 mg L^−1^ and 2 mg L^−1^) where these values were present in the lake. Periods of habitat constraint are indicated along the top of the plot. Generalized additive models show lake whitefish occupancy of (**b**) depth, (**c**) temperature, and (**d**) dissolved oxygen with a global smooth and individual-specific smooths over the study period, with the 20-d periods of interest (May 23–June 11, 2015; July 23–August 11, 2015; September 17–October 6, 2015; and January 23–February 11, 2016) indicated by grey shading

Seasonal changes in the thermal and DO conditions of Lake 658 boundaries (Fig. 3) resulted in dramatic reductions in the amount of usable (< 14 °C and > 2 mg L^−1^ DO) and optimal (< 10 °C and > 4 mg L^−1^ DO) oxythermal habitat available for lake whitefish, both in terms of the percentage of lake volume and percentage of lake bottom (i.e., foraging habitat) (Fig. 2c, d). The loss of lake bottom area was always greater than the reduction in lake volume for a given set of oxythermal habitat constraints. Usable oxythermal habitat in the lake was always present but reached values as low as 10% of lake volume and 6% of lake bottom area in the fall. Optimal oxythermal habitat was absent from the lake for 29 days (Fig. 2c, d). Winter was the season of least habitat constraint, although deep-water hypoxia persisted throughout this period (Figs. 2b, 3).

Generalized additive models fit our data well, explaining between ~ 75 and 95% of the deviance in the data, with the exception of mean total lateral distance and mean lateral speed, which had 42 and 51% of deviance explained, respectively (Supplemental Table 1). These findings demonstrate that similar temporal patterns were present among fish (Fig. 3b–d), but also that individual differences were an important component of the data. For example, in winter, two individuals (LW8 and LW9) were in deeper water than the other three fish, occupying habitat with lower DO concentrations (Fig. 3b, d). With the exception of mean depth, we found that all linear mixed models that included category as a predictor performed better than the null model (i.e., random intercept only) based on the AIC, while the BIC provided similar results with the exception of the model that used DO as a response variable (Supplemental Table 2).

**Fig. 4:**
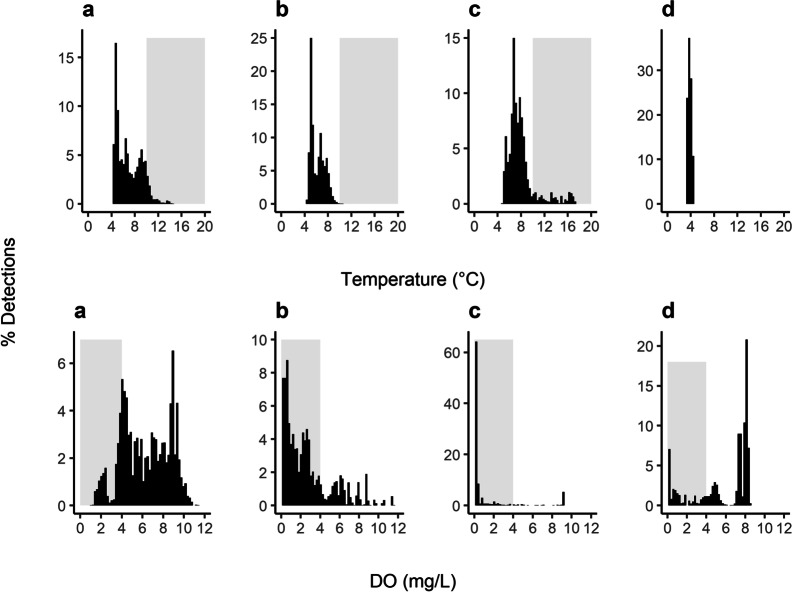
Histograms showing the frequency of detections of acoustically-tagged lake whitefish for twenty consecutive days within each period of oxythermal habitat constraint (**a**—moderate: May 23–June 11, 2015; **b**—high: July 23–August 11, 2015; **c**—severe: September 17–October 6, 2015; and **d**—low: January 23–February 11, 2016). Grey shading highlights habitat with suboptimal conditions (water temperature > 10 °C or dissolved oxygen concentration < 4 mg L^−1^)

**Fig. 5:**
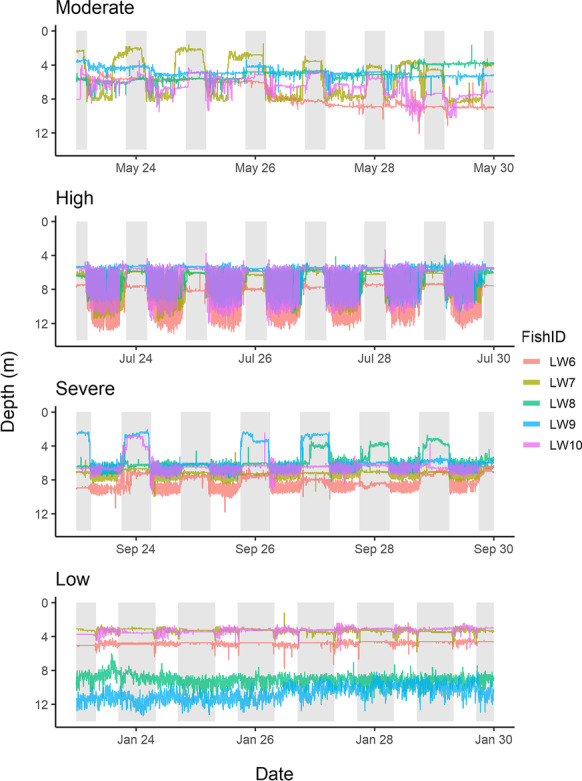
Day (no shading) and night (sunset to sunrise; grey shading) depths occupied by individual lake whitefish (different colours) over a 1-week period (from 00:00:00 CST on the 23^rd^ until 23:59:59 CST on the 29^th^ of May, July, September, and January) during periods of moderate, high, severe, and low oxythermal habitat constraint

During periods of oxythermal habitat constraint, lake whitefish were more often found at depths corresponding to low DO concentrations rather than at depths corresponding to warmer water temperatures. Among the four 20-d periods examined, each representing a different amount of oxythermal constraint, a greater percentage of lake whitefish detections occurred at DO concentrations below 4 mg L^−1^ (51% of all detections in the 80 days) than at temperatures warmer than 10 °C (4%) (Fig. 4). This pattern was exacerbated during the period of severe habitat constraint, where only 11% of detections were in water warmer than 10 °C compared to 91% of detections being in suboptimal oxygen conditions (~ 3% of detections were in suboptimal conditions for both temperature and DO; Table 1). During periods of high and severe habitat constraint (July 23–August 11 and September 17–October 6, 2015), lake whitefish most often (51–87% of detections) occupied hypoxic areas of the lake (< 2 mg L^−1^), whereas no more than 5% of detections were in water warmer than 14 °C in any conditions (Table 1). The use of hypoxic areas was minimal during periods of moderate constraint (May 23–June 12, 2015; 2% of detections), but more frequent (20% of detections) during the period of low oxythermal habitat constraint (January 23–February 11, 2016). The vast majority of detections below 2 mg L^−1^ during low habitat constraint were of two fish (LW8: 8.6%; LW9: 91%) that were frequently at much deeper depths than the other three fish during winter (interquartile range for LW6, LW7, and LW10 = 3.3–4.6 m, interquartile range for LW8 and LW9 = 8.6–11.1 m; Fig. 5). For 8 (LW8) and 20 (LW9) days of this winter 20-d period, these two individuals occupied water at a mean DO of < 4 mg L^−1^.

**Table 1:** The percent of all detections by acoustically-tagged lake whitefish that were within and outside of usable (14 °C and 2 mg L^−1^) and optimal (10 °C and 4 mg L^−1^) oxythermal habitat boundaries over 20 days within each period of constraint

	Usable habitat			Optimal habitat		
Constraint	< 14 °C and > 2 mg L^−1^	> 14 °C	< 2 mg L^−1^	< 10 °C and > 4 mg L^−1^	> 10 °C	< 4 mg L^−1^
Moderate	97.7	0.1	2.3	75.7	8.0	16.4
High	49.0	0.0	50.9	20.6	0.1	79.3
Severe	8.2	4.7	87.1	NA	11.5	91.1
Low	80.0	NA	20.0	75.6	NA	24.4

Over the year-long study period, lake whitefish detections occurred at a mean temperature of 5.8 °C (range = 2.0–21.8 °C) and mean DO concentration of 4.1 mg L^−1^ (range = 0–11.8 mg L^−1^; Fig. 4). Throughout the periods of differing oxythermal habitat constraint, lake whitefish adjusted their depths (mean depths: moderate, 5.9 m; high, 7.4 m; severe, 6.8 m; and low, 6.2 m) to occupy a narrow range of mean water temperatures, especially during the stratified summer period (moderate, 7.0 °C; high, 6.2 °C; severe, 7.9 °C; and low, 3.8 °C). Among the 20-d periods of moderate, high, and severe constraint, the interquartile range of temperatures occupied was 5.3–7.9 °C. Mean DO concentrations at depths occupied by lake whitefish declined as the severity of oxythermal habitat constraint increased (moderate, 6.3 mg L^−1^; high, 2.6 mg L^−1^; severe, 1.1 mg L^−1^; and low, 5.8 mg L^−1^), with an interquartile range of 0.3–5.3 mg L^−1^ DO during the stratified summer period. Thus, during the period of severe habitat constraint, when oxythermal habitat was mostly absent from the lake (Fig. 2), lake whitefish occupied the warmest waters (albeit still < 8 °C) with the least DO compared to the other periods of habitat constraint. Daily mean temperatures differed significantly between habitat categories (χ^2^ = 319, df = 3, *p* < 0.05) and post hoc tests showed that fish occupied lower temperatures during low habitat constraint compared to periods of moderate, high, and severe constraint (*p* < 0.05). Daily mean DO also differed significantly among periods of habitat constraint (χ^2^ = 16, df = 3, *p* < 0.05) with post hoc tests revealing greater occupancy of hypoxic water under high as compared to moderate constraint (*p* < 0.05). Although these conservative models did not show significant differences between other periods of habitat constraint, the coefficient estimates for contrasts between low and severe periods and moderate and severe periods were high relative to the other coefficient estimates (i.e., > 1).

The vertical distribution of lake whitefish encompassed the entire lake, with detections from depths of 0.1 to 14.0 m. During the 20-d periods representing a gradient of oxythermal habitat constraint, fish depths ranged from 0.6 to 13.9 m, with no significant differences between periods of habitat constraint (χ^2^ = 6, df = 3, *p* = 0.1). However, there was considerable difference among individual patterns in depths occupied. As an example of fine-grained behavioural changes, we present the depth distributions of individual tagged lake whitefish for a period of 1 week in May, July, and September 2015 and January 2016, each of which comprise part of the 20-d periods of moderate, high, severe, and low oxythermal habitat constraint, respectively (Fig. 5). The periods of low and moderate constraint, with a larger range of depths, corresponded to greater inter-individual variation in depth distribution (Fig. 5).

**Fig. 6:**
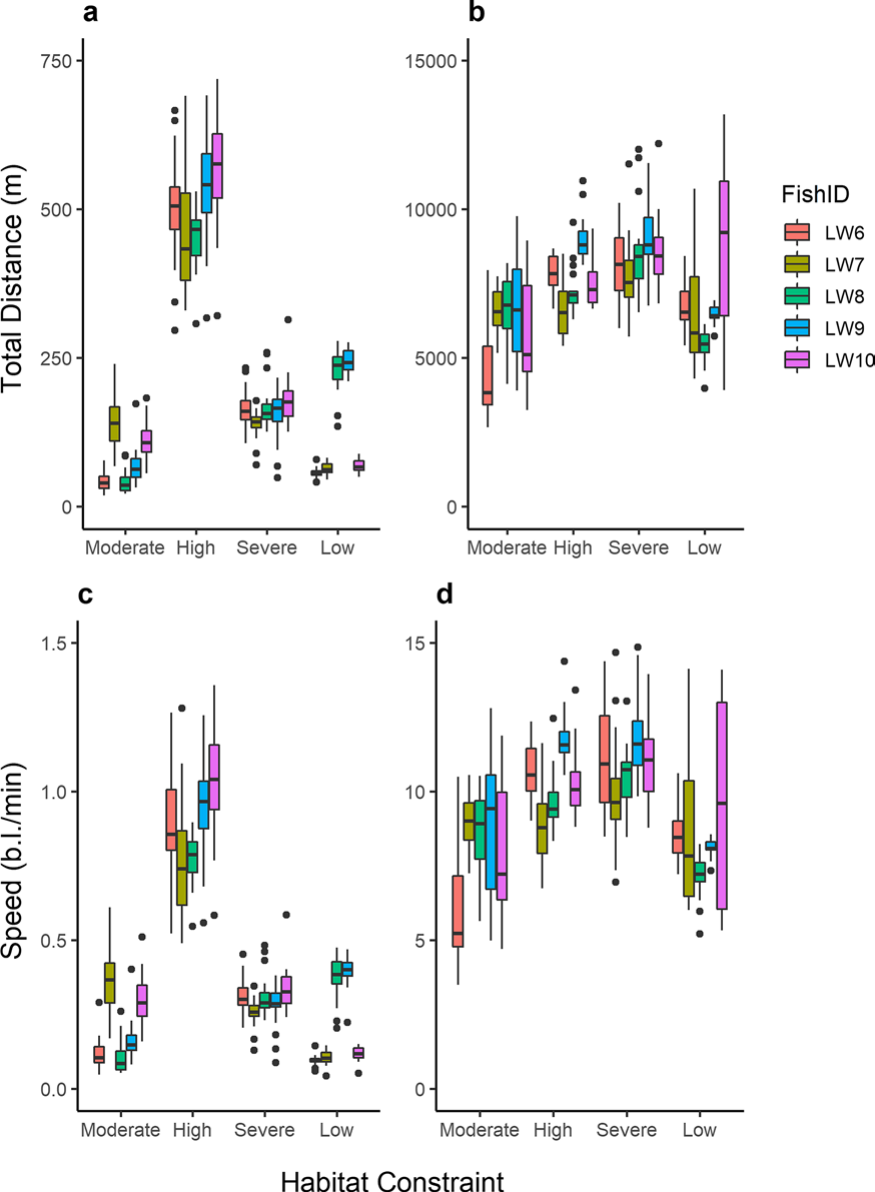
Box plot showing the total (**a**) vertical and (**b**) lateral daily mean total distance, and (**c**) vertical and (**d**) lateral daily mean swim speeds (body length min^−1^) for lake whitefish for the 20-day subsets for each period of habitat constraint (May 23–June 12, 2015; July 23–August 11, 2015; September 17–October 6, 2015; and January 23–February 11, 2016). The top and bottom of each box represent the first and third quartiles, and the median line is shown. Whiskers show data within 1.5 times the interquartile range of the nearest quartile and outliers are plotted as individual points

Mean total vertical distances differed significantly (χ^2^ = 230, df = 3, *p* < 0.05) between all pair-wise comparisons of habitat constraint (*p* < 0.05) except low and moderate (*p* = 0.17; Fig. 6a). Under high oxythermal constraint, when the mean depth of lake whitefish was deepest, these fish had the highest rate of total vertical movement with a mean of 502.5 m per day up and down in the water column compared to 81.2, 160.9, and 131.9 m in periods of moderate, severe, and low constraint, respectively (Figs. 5, 6a). This corresponds to the pattern of repeated vertical forays (Fig. 5). During the period of high oxythermal habitat constraint, the vertical swim speed averaged 0.88 body lengths per minute (b.l. min^−1^), compared to 0.21, 0.30, and 0.22 b.l. min^−1^ in periods of moderate, severe, and low constraint, respectively. Daily mean vertical swim speeds were significantly different (χ^2^ = 212, df = 3, *p* < 0.05) between all pair-wise comparisons (*p* < 0.05) except between low and moderate constraint (*p* = 0.99; Fig. 6b).

The lowest rate of vertical movement by whitefish (mean total distance per day = 81 m, mean swim speed = 0.21 b.l. min^−1^) occurred during the period of moderate constraint, which was also the period of least lateral movement (mean total distance per day = 6109 m; Fig. 6b). This period of decreased movement on both planes corresponded with a lack of consistent diel or inter-individual patterns in depth distribution by lake whitefish (Fig. 5). Total lateral distances moved differed between periods of constraint (χ^2^ = 49, df = 3, *p* < 0.05); specifically, post hoc tests showed differences between moderate and severe constraint, moderate and high constraint, and low and severe constraint (*p* < 0.05). Lateral distances were greatest under severe constraint (mean = 8392 m), followed by high (mean = 7649 m), low (mean = 6782 m), and moderate constraint (Fig. 6b). Daily mean lateral swim speeds were also significantly different among categories (χ^2^ = 45, df = 3, *p* < 0.05), including all pairwise comparisons except for high and severe habitat constraint (*p* = 0.27) and low and moderate habitat constraint (*p* = 0.81). Differences followed the same pattern as total lateral distances, with the fastest swim speeds under severe constraint (mean = 11.11 b.l. min^−1^), then high (mean = 10.24 b.l. min^−1^), followed by low (mean = 8.90 b.l. min^−1^) and moderate (mean = 8.16 b.l. min^−1^) constraint (Fig. 6d).

**Fig. 7:**
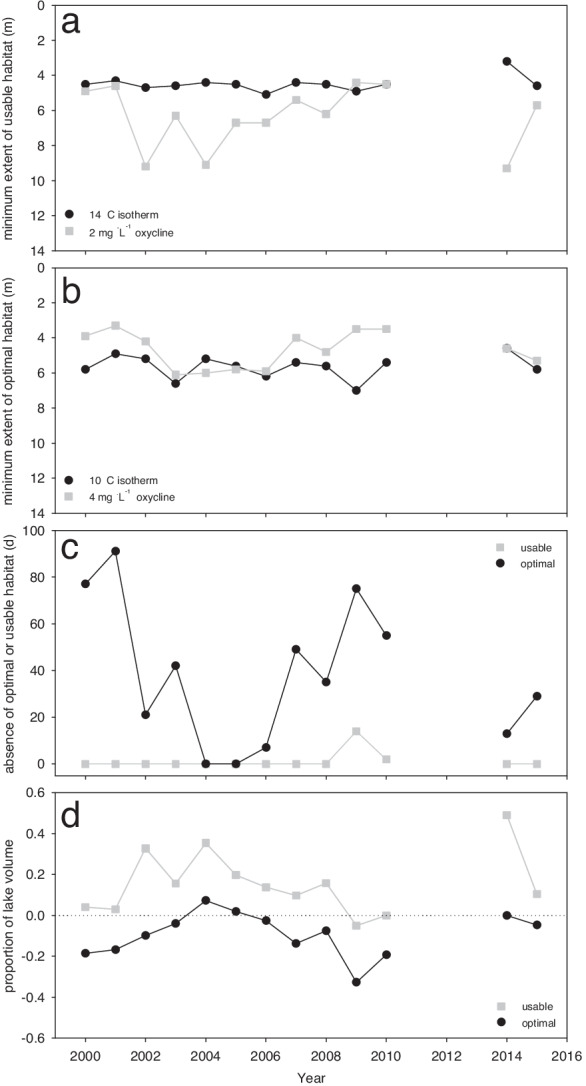
The depth within Lake 658 at which oxythermal habitat boundaries reached an annual maximum constraint on (**a**) usable habitat (14 °C isotherm and 2 mg L^−1^ oxycline) and (**b**) optimal habitat (10 °C isotherm and 4 mg L^−1^ oxycline) for lake whitefish. Instances where the oxyclines (grey squares) are found at the same or shallower depths than the isotherms (black circles) represent the complete loss of  optimal/usable oxythermal habitat in that year. The number of days where optimal/usable oxythermal habitat is absent from the lake is shown in panel (**c**). The proportion of total lake volume that is usable (grey) and optimal (black) at the point of maximum habitat constraint each year is shown in panel (**d**). Positive values indicate the proportion of the lake in which both temperature and oxygen conditions are optimal/usable. Negative values indicate that the entire lake volume is void of optimal/usable oxythermal habitat based on at least one parameter, with the negative value representing the proportion of the lake in which both temperature and oxygen parameters are suboptimal/unusable. Temperature and oxygen data were not collected in 2011–2013

Analysis of historical temperature and DO data show that habitat pressures are chronically severe in Lake 658 (Fig. 7). At the time of annual minimum habitat availability, both the 10 °C and 14 °C isotherms are routinely deeper than 4 m, while the 4 mg L^−1^ oxycline is routinely shallower than 6 m (Fig. 7a, b). We estimated a complete absence of optimal lake whitefish habitat for > 20 days per year in at least 9 years from 2000 to 2015 (Fig. 7c), and in one of the most severe years of habitat constraints (2009), all usable habitat for whitefish was absent from Lake 658 for 14 days. In years when the lake became void of optimal habitat, large proportions of the lake volume (up to 32.7%; see negative values in Fig. 7d) were suboptimal for both thermal and DO criteria, with the remaining lake volume being suboptimal for only one of the two parameters.

## Discussion

Lake whitefish typically inhabit deep lakes with cold, well-oxygenated water that provides access to benthic habitat in which to forage. The regular occurrence of deepwater hypoxia in Lake 658 provided a unique opportunity to examine the habitat choices of a natural lake whitefish population that is simultaneously subjected to severe temperature and DO constraints. Optimal oxythermal habitat for lake whitefish in Lake 658 was constrained for the duration of the study due to the persistence of deep-water hypoxia throughout the entire year. A steady deepening of the upper thermal boundaries for lake whitefish with the progression of summer stratification occurred as hypoxic waters expanded upwards from the lake bottom and resulted in the greatest reductions in available oxythermal habitat from August to mid-October. During this time, lake whitefish predominantly occupied hypoxic areas of the lake (> 90% of detections at < 4 mg L^−1^ DO) and were rarely detected (< 15% detections) in water warmer than 10 °C. Seasonal changes in oxythermal habitat influenced activity patterns of lake whitefish, with distinct changes in vertical and horizontal movements associated with periods of high and severe habitat constraints. Collectively, our findings show that lake whitefish exhibit behavioural changes and individual strategies to cope with the severe and chronic exposure to suboptimal habitat conditions.

Lake whitefish were detected in water temperatures ranging from 2 to 22 °C over the course of an entire year; however, they were mainly found at a very narrow range of temperatures (interquartile range = 5.3–7.9 °C) during the stratified period. In a previous telemetry study in Clear Lake, Maine (253 ha), lake whitefish were found to occupy water temperatures of 10–16 °C in summer (Gorsky et al. 2012), consistent with the habitat niche of 10–14 °C defined by Christie and Regier (1988). The much colder water temperatures occupied by lake whitefish in the present study are more consistent with those of habitat occupancy models derived from netting surveys in larger lakes in Algonquin Park (649 ha and 5860 ha), Canada, where high occupancy occurred at temperatures of 6.2–7.6 °C and 7.7–13.6 °C during the stratified period in the smaller and larger study lakes, respectively (Challice et al. 2019). There was no evidence of hypoxia at the depths occupied by lake whitefish in the Algonquin Park study lakes (Challice et al. 2019), and therefore temperature occupancy was not influenced by avoidance of hypoxic bottom waters in these lakes.

We found that lake whitefish will avoid temperatures greater than 10 °C even if this behaviour exposes them to hypoxic conditions. Across all periods of oxythermal constraint, lake whitefish were detected more frequently in hypoxic conditions (< 2 mg L^–^^1^ DO) than in warm water (> 14 °C), with an interquartile range of 0.3–5.3 mg L^–^^1^ DO during summer stratification. While the range of temperatures occupied was predominantly within optimal thermal habitat, the range of DO conditions occupied was often well below limits considered as the lower optimal and usable habitat boundaries for other coldwater salmonids (Plumb and Blanchfield 2009; Jacobson et al. 2010). Lake whitefish used hypoxic areas throughout most of the year, driven both by thermal pressure during periods of high and severe oxythermal constraint and by individual habitat preferences in winter. More than 75% of whitefish detections occurred in suboptimal oxygen conditions during periods of high and severe constraint, and over half of these detections occurred in < 2 mg L^−1^ DO, which is lower than the < 3 mg L^–^^1^ threshold considered unusable habitat for coregonid species (Jacobson et al. 2010). During low habitat constraint, 20% of detections were in water < 2 mg L^–^^1^, although all of these detections were of two fish that consistently occupied deep areas of the lake in winter, suggesting individual habitat preferences among whitefish. Under moderate constraint, when 10–50% of the total lake volume satisfied optimal habitat requirements, all individuals occupied a similar range of depths, such that 76% of detections were in optimal habitat and 98% were in usable habitat. Together, these results indicate that lake whitefish vertical distributions were largely contained within the oxythermal boundaries we defined as optimal (< 10 °C and > 4 mg L^−1^ DO) and usable (< 14 °C and > 2 mg L^−1^ DO) when this habitat was available. When optimal oxythermal habitat was limited, lake whitefish selected hypoxic waters and could tolerate DO < 2 mg L^−1^ for extended periods of time.

Lake whitefish showed striking differences in movement patterns as oxythermal conditions changed. We observed the greatest amount of vertical movement, averaging ~ 500 m per fish per day, during high oxythermal habitat constraint (Fig. 6a), where the available lake bottom area was 1–10% of the total lake. Fish were mostly constrained within a ~ 3 m band (between 5.9 and 8.6 m) in the water column at this time, so this distance is roughly equivalent to 93 vertical forays each day, almost all of which would have occurred during daylight hours (Fig. 5). During this period, lake whitefish were largely detected in the hypoxic region of the lake but still had access to optimal oxythermal habitat. Optimal habitat may have provided refuge for recovery from the physiological stress of low oxygen, thereby allowing for a high number of vertical forays to forage for hypoxia-tolerant benthic chironomids and chaoborids. Similar explanations have been proposed for other fishes’ use of hypoxic habitat when refuge is available, including for yellowtail kingfish (*Seriola lalandi* Valenciennes 1833; Cook and Herbert 2012) and Atlantic cod (*Gadus morhua* Linnaeus 1758; Herbert et al. 2011). The mean daily distance moved vertically in the water column was sixfold and threefold greater than the preceding and following periods of moderate and severe habitat constraint, respectively. This finding suggests that the mid-summer period may be especially critical for lake whitefish foraging, when some optimal habitat remains and when they may be driven by high energy needs due to warmer summer temperatures (Reynolds and Casterlin 1980).

The horizontal distances travelled by lake whitefish were also highly variable across the gradient of oxythermal constraints encountered in Lake 658. Interestingly, daily horizontal movement was greatest (mean = 8392 m) during the period of severe habitat constraint, although lake whitefish had a narrower depth distribution (interquartile range = 6.2–7.3 m) and travelled up and down much less (daily total mean = 161 m) during this period than during the preceding period of high constraint. The finding that whitefish lateral movement was greatest during the harshest conditions, an observation that was also supported by post hoc analysis of our linear mixed model, suggests that they adopted a different strategy to access sufficient prey. In particular, if pelagic prey were abundant, this would be consistent with previous findings of optimization of foraging strategy relative to seasonal prey distributions (Gorsky et al. 2012). Additionally, whereas vertical movement under high constraint presumably allowed for foraging forays into suboptimal habitats and subsequent recovery from those stressful conditions, habitat was sufficiently limited under severe constraint that respite from suboptimal oxythermal conditions was not possible. Overall activity may have been similar to the period of high constraint due to the increase in lateral movement, but forced into a much narrower plane (Fig. 5) to account for the physiological stress of severe oxythermal conditions (mean DO at detection = 1.1 mg L^−1^, mean temperature at detection = 7.9 °C), potentially resulting in suboptimal rates of energy acquisition.

The spring and early summer period of this study represented a time in which usable oxythermal habitat was abundant in the study lake (the period of moderate constraint; May to late-June). At this time, almost all detections (98%) of lake whitefish were contained within usable habitat boundaries. Vertical and lateral movements were at their lowest under these conditions, suggesting that the greater activity observed during high and severe oxythermal constraints were strategies used to access prey in the period leading up to spawning. The period of moderate habitat constraint also corresponded to a period of high inter-individual and diel variation in movement behaviour (Fig. 5). Woodard et al. (2021) found the highest level of individual specialization in the spring (March–May) in their study of lake whitefish diets off the Keweenaw Peninsula of Lake Superior, and Gorsky et al. (2012) suggested that the increased diel vertical migration and shallower depth occupancy in spring that they observed in lake whitefish in Clear Lake is driven, in part, by prey availability. In our study, moderate constraint likely afforded the greatest flexibility in prey use, given favourable thermal conditions coinciding with a spring increase in lake productivity and invertebrate emergence.

Altogether, activity levels of lake whitefish in the winter were low. Both vertical and lateral distances travelled were higher than under moderate constraint but lower than under high and severe constraint (vertical daily total mean = 132 m, lateral daily total mean = 6782 m). Previous research on lake trout has found similar levels of activity between winter and summer (Blanchfield et al. 2009b), but winter is expected to be a period of high divergence in activity rates among species (McMeans et al. 2020). While the overall range of depths was high (interquartile range = 3.4–9.1 m), this was influenced by the occurrence of two distinct activity patterns in the study population. The apparent separation into shallow and deep habitat occupancy and coincident lake whitefish activity patterns observed during winter may represent different foraging strategies, as the shallower fish maintained a diel pattern in their vertical movement while the deeper fish, in hypoxic conditions, maintained vertical movements throughout the day and night (Fig. 5). Inter-individual differences in movement behaviour have been found in many other fishes, including another species of coregonid, European whitefish (*Coregonus lavaretus* Linnaeus 1758, Ovidio et al. 2006), and other salmonids such as lake trout (Blanchfield et al. 2009b), bull trout (*Salvelinus confluentus* Suckley 1859, Taylor and Cooke 2014), and rainbow trout (*Oncorhynchus mykiss* Walbaum 1792, Watson et al. 2019). The lifting of thermal constraints in winter may promote individual specialization in foraging, especially for coldwater species that continue to be active during winter (McMeans et al. 2020). Variation in habitat selection by individuals may affect both the availability of prey and the extent of physiological stress, which in turn may lead to greater variation in growth rates and body condition (Roberts et al. 2009).

The prolonged use of hypoxic waters by lake whitefish observed in this study has not previously been documented, to our knowledge, and runs counter to the general avoidance of hypoxia exhibited by other fish species. For example, the closely-related cisco are restricted by bottom hypoxia (Aku et al. 1997), and lake trout in small boreal lakes near Lake 658 more consistently occupy highly oxygenated water than their optimal thermal habitat (Sellers et al. 1998; Plumb and Blanchfield 2009). Lake trout are known to make excursions into thermally suboptimal habitat (Morbey et al. 2006; Guzzo et al. 2017) where they are likely to benefit energetically from larger prey (Plumb et al. 2014; Guzzo et al. 2017). As benthivores, the energetic benefit of using benthic habitat may outweigh the costs of withstanding hypoxia for lake whitefish. Yellow perch, another benthivore present in this lake, avoid hypoxia in Lake Erie but will foray into low oxygen conditions when hypolimnetic prey quality is sufficiently high (Roberts et al. 2009). Nati et al. (2018) suggested that the benthic species European bullhead (*Cottus gobio* Linnaeus 1758) and stone loach (*Barbatula barbatula* Linnaeus 1758) may choose hypoxic habitat with higher prey density and protection from predators over normoxic habitat with less food and more risk of predation. In shallow lakes with severe oxythermal habitat limitations, up to and including the complete loss of optimal habitat, benthivores may benefit energetically from repeated forays into hypoxia and thus withstand the potential physiological cost.

Suitable oxythermal habitat is predicted to decrease over time as temperate lakes face increased warming and lower levels of DO driven by climate change (Jane et al. 2021; Woolway et al. 2022). Lake whitefish in this study population have faced annual constraints in the amount of optimal oxythermal habitat for 11 of the 13 years on record from 2000 to 2015, ranging from 7 to 91 days per year, including an extreme year (2009) when even usable habitat (i.e., < 14 °C and > 2 mg L^−1^ DO) was not available in the lake for 2 weeks. During the period of lake whitefish habitat monitoring in Lake 658, tagged fish consistently avoided water warmer than 10 °C. Based on the long-term monitoring data, the maximum depth of the 10 °C isotherm was > 4 m, limiting optimum habitat availability to the bottom 9 m of the lake, of which up to 6 m may be hypoxic. Loss of optimal oxythermal habitat for lake trout (< 10 °C and > 4 mg L^−1^ DO) is also prevalent in four nearby lakes (Guzzo and Blanchfield 2017). If temperatures of lakes currently supporting lake whitefish populations continue to increase, populations may be extirpated by increasingly deep thermoclines, as has already begun near the southern edge of their range (Renik et al. 2020). The lake whitefish in this study showed remarkable tolerance of hypoxic conditions, which allowed them to use optimal thermal habitat even under severe oxythermal constraint. Their ability to withstand hypoxic habitat may allow them to survive the 1–2 mg L^−1^ decrease in DO predicted for temperate lakes by Blumberg and Di Toro (1990) and Missaghi et al. (2017). However, limited benthic access due to high littoral temperatures during summer stratification and extended bottom hypoxia may drastically limit their access to high-quality prey, and changes in foraging behaviour due to reductions in oxythermal habitat may reduce foraging efficiency as well.

Adult lake whitefish spawn in shallow nearshore regions in the fall and feed on zooplankton in this region as larvae in the spring (Pothoven 2020). Increased periods of stratification and lack of optimal oxythermal habitat in the late fall may delay access to spawning grounds for lake whitefish and other fall spawners, altering their life history phenology (Missaghi et al. 2017). A long-term study of four lakes near to our study site found that the duration of stratification is not increasing, but that it is shifting to later in the year following the increasing length of the spring period where surface water temperatures are 4–15 °C (Guzzo and Blanchfield 2017). This shift has delayed the timing of lake trout spawning by 10 days compared to the 1970s (Guzzo and Blanchfield 2017). If shifting stratification periods and windows of optimal oxythermal habitat also affect lake whitefish spawning, this could reduce the time available for egg development before spring hatching. It could also lead to phenological mismatches such as larvae developing later than the emergence of sufficient zooplankton prey (Wong and Candolin 2015). Discrepancies between the lengths of life history stages, habitat availability, and prey availability may lead to low early-stage survival and low recruitment rates.

In summary, lake whitefish exhibited seasonal and inter-individual variability in movement and habitat selection in response to the availability of optimal oxythermal habitat. This population maintained a thermal distribution largely within 5.3–7.9 °C during the stratified period, which agrees with our expectation for a coldwater species. However, they consistently spent time in suboptimal oxygen habitat when warm epilimnetic temperatures precluded the use of well-oxygenated habitat, indicating that avoidance of warm water had a greater impact on their distribution than avoidance of hypoxia. As climate-driven changes in thermal and oxygen conditions reduce or eliminate the availability of lake whitefish habitat, both physiological stress—including that of persistent exposure to hypoxia—and potential resultant changes in foraging strategies across life stages may limit spawning success, recruitment, and overall population size. Encouragingly, however, the fish in this study showed remarkable tolerance of long-term hypoxia, which suggests an inherent ability of lake whitefish to withstand what were previously considered unusable oxythermal conditions in the wild.

## Supplementary Information

Below is the link to the electronic supplementary material.

Supplementary file1 (DOCX 18 KB)

## Data Availability

Data collected and discussed in this study are available upon request to IISD Experimental Lakes Area: https://www.iisd.org/ela/science-data/our-data/data-requests/.
