# Supplementary material for: Behavioural responses of a cold-water benthivore to loss of oxythermal habitat
Source: Environ Biol Fishes. 2022 Sep 28;105(10):1489–507. doi: 10.1007/s10641-022-01335-4 (PMC9592630; doi:10.1007/s10641-022-01335-4)
Supplement: Supplementary file 1 — Supplementary file1 (DOCX 18 KB) [file 10641_2022_1335_MOESM1_ESM.docx]

**Supplemental Table. 1.** Abbreviated results from Generalized Additive Models. All models took the form: g(*µ_i_*) = *b_j_* + ƒ(julian*_i_*) + ƒ*_j_* (julian*_i_*), where g(*µ_i_*) is an inversible monotonic link function, *b_j_* is the intercept for the *j*^th^ individual, ƒ(julian*_i_*) is the global smooth of julian day, and ƒ*_j_* (julian*_i_*) represents the smooth of julian day for the *j*^th^ individual.

| **Response variable** | **% Deviance explained** | **Estimated total degrees of freedom** |
| --- | --- | --- |
| mean depth | 83.7 | 79 |
| mean temperature | 96.0 | 78 |
| mean dissolved oxygen | 85.9 | 65 |
| total lateral distance | 42.4 | 60 |
| total vertical distance | 77.7 | 64 |
| mean lateral speed | 51.2 | 58 |
| mean vertical speed | 78.6 | 64 |

**Supplemental Table. 2.** Results from comparison of a null model (random intercept for Fish ID only) and the model that included stress category as an explanatory variable (“full model”).

| **Response variable** | **Model type** | **Degrees of freedom** | **AIC** | **BIC** |
| --- | --- | --- | --- | --- |
| mean depth | *Null* | 7 | -1232 | -1203 |
|  | *Full* | 10 | -1232 | -1192 |
| mean temperature | *Null* | 7 | -1470 | -1442 |
|  | *Full* | 10 | -1522 | -1482 |
| mean dissolved oxygen | *Null* | 7 | -21 | 7 |
|  | *Full* | 10 | -29 | 11 |
| mean lateral distance | *Null* | 7 | -192 | -164 |
|  | *Full* | 10 | -221 | -181 |
| mean vertical distance | *Null* | 7 | 181 | 209 |
|  | *Full* | 10 | 120 | 160 |
| mean lateral speed | *Null* | 7 | -224 | -196 |
|  | *Full* | 10 | -250 | -210 |
| mean vertical speed | *Null* | 7 | 209 | 237 |
|  | *Full* | 10 | 148 | 188 |
